# Blocking α_2_δ-1 Subunit Reduces Bladder Hypersensitivity and Inflammation in a Cystitis Mouse Model by Decreasing NF-kB Pathway Activation

**DOI:** 10.3389/fphar.2019.00133

**Published:** 2019-02-26

**Authors:** Ludivine Boudieu, Sarah Mountadem, Amandine Lashermes, Mathieu Meleine, Lauriane Ulmann, François Rassendren, Youssef Aissouni, Benoit Sion, Frédéric Antonio Carvalho, Denis Ardid

**Affiliations:** ^1^NEURO-DOL, Université Clermont Auvergne, Clermont-Ferrand, France; ^2^U1107, Inserm, Clermont-Ferrand, France; ^3^IGF, CNRS, INSERM, Univ Montpellier, Montpellier, France; ^4^Labex ICST, Montpellier, France

**Keywords:** cystitis mouse model, α_2_δ-1 ligands treatment, pain, inflammation, NF-kB pathway

## Abstract

Bladder pain is frequently associated with bladder inflammation, as in conditions like interstitial cystitis (IC), for which current analgesic therapies have limited efficacy. The antinociceptive effect of alpha-2-delta (α_2_δ) ligands on inflammation-associated visceral pain like that experienced in cystitis has been poorly investigated. To investigate the effect of pregabalin (PGB), an α_2_δ ligand, we evaluated its impact on mechanical hyperalgesia in a mouse model of cystitis induced by cyclophosphamide (CYP). We further studied its effect on inflammation and NF-kB pathway activation. Acute cystitis was induced by intraperitoneal injection of 150 mg kg^-1^ of CYP in C57Bl/6J male mice. PGB was subcutaneously injected (30 mg kg^-1^) 3 h after CYP injection. The effect of PGB on CYP-induced mechanical referred hyperalgesia (abdominal Von Frey test), inflammation (organ weight, cytokine production, α_2_δ subunit level, NF-kB pathway activation) were assessed 1 h after its injection. In parallel, its effect on cytokine production, α_2_δ subunit level and NF-kB pathway activation was assessed *in vitro* on peritoneal exudate cells (PECs) stimulated with LPS. PGB treatment decreased mechanical referred hyperalgesia. Interestingly, it had an anti-inflammatory effect in the cystitis model by reducing pro-inflammatory cytokine production. PGB also inhibited NF-kB pathway activation in the cystitis model and in macrophages stimulated with LPS, in which it blocked the increase in intracellular calcium. This study shows the efficacy of PGB in hypersensitivity and inflammation associated with cystitis. It is therefore of great interest in assessing the benefit of α_2_δ ligands in patients suffering from cystitis.

## Introduction

Bladder pain is frequently associated with bladder inflammation, as in interstitial cystitis (IC) (Ogawa et al., 2015). The mechanisms include an increase in mucosal bladder permeability (Buffington and Woodworth, 1997), leading to sensitization of bladder afferent pathways and inflammation (Yoshimura et al., 2002), and sensitization of peripheral and/or central pain pathways (Ogawa et al., 2015). At the periphery, inflammatory processes could be involved. Increased levels of pro-inflammatory cytokines (Erickson et al., 2002) and a decrease in those of the anti-inflammatory IL-4 cytokine (Ueda et al., 2000) have been observed in IC patients. The efficacy of anti-nerve growth factor (NGF) therapy in humans (Evans et al., 2011) confirms the involvement of NGF in the pathophysiology of bladder pain. All these peripheral mediators can sensitize the mechanosensitive afferent fibers or increase their recruitment (Sengupta and Gebhart, 1994).

The existing treatments of IC involve non-pharmacological approaches such as behavioral modifications, bladder hydrodistention, neurostimulation, and surgery. Pharmacological treatments are divided into two categories, peripheral (i.e., intravesical) and systemic. The intravesical treatments include dimethyl sulfoxide, heparin, lidocaine, and onabotulinum toxin A (Kuo and Chancellor, 2009; Ogawa et al., 2015). Systemic pharmacological therapies include amitriptyline, a tricyclic antidepressant used in the first line therapy for neuropathic pain to improve symptoms associated with IC such as pain and urgency (van Ophoven et al., 2004).

Anticonvulsants are used in the treatment of several chronic pain syndromes, targeting neuronal excitability (Johannessen Landmark, 2008). Among these, the α_2_δ ligands, gabapentin (GBP) and pregabalin (PGB) act on voltage-gated calcium (Ca^2+^) channels (VGCCs) to inhibit pre-synaptic glutamate release (Johannessen Landmark, 2008). In addition, previous studies have demonstrated potent and selective binding of PGB to α_2_δ-1 and α_2_δ-2 subunits (Li et al., 2011). These molecules are now proposed in first line with antidepressant drugs for treatment of neuropathic pain (Johannessen Landmark, 2008). Their beneficial effect has been observed in cystitis patients (Hansen, 2000; Sasaki et al., 2001). However, some preclinical studies failed to find any effect of GBP in rodent cystitis models (Rudick et al., 2009). Our study aimed to investigate the potential beneficial effects of PGB, an α_2_δ ligand, on a mouse model of cystitis induced by cyclophosphamide (CYP). It has been reported that α_2_δ ligands reduce the activation of the nuclear factor kB (NF-kB) in neuroblastoma and glioma cells (Park et al., 2008). In light of these findings, we further investigated the mechanism of action of α_2_δ ligands by studying their interaction with NF-kBpathway activation.

## Materials and Methods

### Acute Cystitis Induction

All experiments were performed according to the ethical guidelines set out in the Guide for the Care and Use of Laboratory Animals and with approval of the “Comité d’Ethique pour l’Expérimentation Animale Auvergne” (C2E2A), the local ethics committee (Reference number: EU0116-5330). All experiments were performed on C57Bl/6J male mice weighing 20–24 g (JANVIER LABS, Le Genest Saint Isle, France). Animals were given access to food and water *ad libitum* and housed with a 12-h light-dark cycle. Acute cystitis was induced by intraperitoneal injection of 150 mg kg^-1^ of CYP. Control mice received saline injection.

### Pregabalin Treatment

Pregabalin ((S)-((+)-3-(aminomethyl)-5-methylhexanoic acid; Dochem lot PRE20110601) was dissolved in 0.9% saline. 3 h after CYP injection, mice were subcutaneously injected with PGB (30 mg kg^-1^) or saline. Tests were performed 1 h later.

### Mechanical Referred Hyperalgesia Testing

Mechanical cutaneous abdominal sensitivity was assessed with von Frey filaments (Biosed, Vitrolles, France) before the animals were injected and 4 h after CYP injection. The filaments were applied to the lower abdominal area close to the urinary bladder and the median 50% threshold (T50) was determined by the up-and-down method (Chaplan et al., 1994). Briefly, this method is based on the use of the 3.22 filament size (0.16 g), which corresponds to the intermediate size of the filament range. The filament is applied perpendicular to the lower abdominal area close to the urinary bladder, exerting sufficient force to flex it for a period of 5 s. Two answers were possible: (i) animal reacts (abdominal contraction), this response is marked “X” and the test continues with the smaller filament on the range (2.83/0.07 g); (ii) animal does not respond, this response is marked “O” and the test continues with the larger filament in the range (3.61/0.4 g). This scheme continues until the objectivized response of the animal changes compared to the first reaction observed with the 3.22 filament size. From that moment, four filaments are applied (always according to the same method) and the test is finished. The pattern thus obtained corresponds to a score available in the appendix of the Chaplan publication (Chaplan et al., 1994). Finally, thanks to the Dixon formula (Dixon, 1980), it possible to calculate the median 50% threshold (T50): (10 ^[Xf+κδ]^) / 10000, with Xf, size of the last applied filament, κ, score and δ, average difference between stimuli.

### Bladder Culture

Following euthanasia, the bladder was removed, cut open longitudinally, washed in PBS and cultured in RPMI1640 medium containing penicillin and streptomycin. After 24 h incubation at 37°C with 5% CO_2_, supernatants were centrifuged at 4°C and used for assaying cytokines by ELISA.

### Enzyme-Linked Immunosorbent Assay

All ELISA kits are DuoSet kits from R&D Systems, and assays were performed according to the manufacturer’s protocol.

### Tissue Myeloperoxidase Assay

One-third of the bladder was homogenized (50 mg mL^-1^) in 0.5% hexadecyltrimethylammonium bromide (Sigma) in 50 mM PBS, (pH 6.0), freeze-thawed three times, sonicated and centrifuged. Myeloperoxydase (MPO) was assayed in the supernatant by adding 1 mg mL^-1^ of O-dianisidine dihydrochloride (Sigma) and 5 × 10–4% H_2_O_2_. One unit of MPO activity was defined as the amount that degraded 1.0 μmol of peroxide/min at 25°C.

### Histology

Bladder domes were fixed for 24 h in 4% buffered formalin at 4°C and then subjected to Hematoxylin and Eosin staining on 5 μm thick tissue sections. The mucosal thickness was measured with Gimp 2.8 software. Four measured per sections and three sections per animal were assessed.

### Western Blotting

Four hours after CYP injection, mice were euthanized and their bladders collected. For IκBα, phospho-p65 and α2δ-1 total expression study, bladders were homogenized in ice-cold lysis buffer containing stop buffer and protease inhibitor cocktail. For determination of α2δ-1 membrane/cytoplasmic expression, proteins were extracted according to the manufacturer’s protocol for use of the Compartmental Protein Extraction Kit (Merck Millipore). Blotting was performed at 4°C overnight with the following antibodies: IκBα (1:500; Santa Cruz Biotechnology), phospho-p65 (Ser276; 1:500; Santa Cruz Biotechnology), α2δ-1 (1:500; Santa Cruz Biotechnology), phospho-ERK1/2 (T202/Y204; 1:1000; Cell signaling Technology), ERK1/2 (1:1000; Cell Signaling Technology), EGFR (1:1000; Santa Cruz Biotechnology), and β-actin (Sigma-Aldrich). Protein quantification was performed by densitometry using ChemiDoc MP imager and Image Lab^TM^ software (Bio-Rad).

### *In vitro* Peritoneal Exudate Cell Studies

Resident mouse peritoneal exudate cells (PECs) were collected from euthanized animals. The abdominal skin was incised for reveal the abdominal muscle. Two abdominals lavage were successively realized with 5 ml of PBS+0.5% of fetal bovine serum (FBS). The cells thus collected were count on Malassez cell, centrifuged (300 *g*, 8 min, 4°C) and resuspended in DMEM (+10% FBS and seeded at the density of 4 × 10^5^ cells/well). After overnight incubation, cells were co-incubated with LPS (100 ng mL^-1^; Sigma-Aldrich Chimie, Saint-Quentin-Fallavier, France) and PGB (11.3 μM) or saline inserum-free media.

### Intracellular Calcium Imaging and Measurement

Cells (PECs) were loaded with 2 μM of Fura-2-acetoxymethyl ester (Fura-2/AM, Life Technologies), 0.5% BSA in the recording saline solution. After 1 h, cells were stimulated for 2 min with LPS (1 μg/mL). When indicated, cells were also pre-incubated with PGB (11.3 μM) for 15 min. Intracellular Ca^2+^ concentration ([Ca^2+^]) was assessed by recording the changes in cytoplasmic [Ca^2+^] with the ratiometric fluorescent probe Fura-2 in PECs. The MetaFluor Imaging System (Molecular Devices) was used for fluorescence acquisition and analysis of individual cells. Pairs of images were acquired every 2 s. A single PEC was considered as a responder if the F340/F380 ratio for a single PECincreased by 0.05.

### Statistical Analysis

All data were expressed as mean ± SEM and analyzed with GraphPad Prism5 software. Differences in T50 and the effects of PGB on *ex vivo* parameters were analyzed by a 1-way ANOVA (Treatment) followed by Tukey *post hoc* test for multiple comparisons. The effects of PGB on IκBα, phospho-p65 and α2δ-1 expression in PECs culture were analyzed by a 2-way ANOVA (Model, Treatment) followed by Bonferroni *post hoc* test for multiple comparisons. For calcium imaging experiments, statistical differences were elicited by a Mann–Whitney *U*-test. A *p*-value less than 0.05 was consideredstatistically significant.

## Results

### Effect of Pregabalin on Cutaneous-Referred Bladder Hypersensitivity in Acute Cyclophosphamide-Induced Cystitis

**FIGURE 1:**
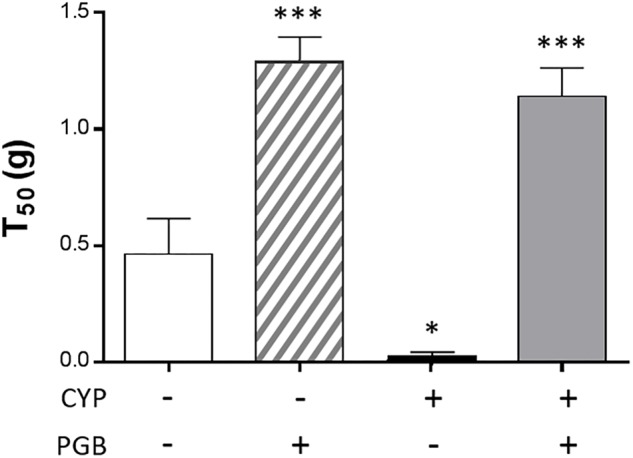
Effect of pregabalin (PGB; 30 mg kg^-1^, s.c.) on cystitis-induced mechanical referred hyperalgesia assessed by the abdominal von Frey test. The von Frey filaments were applied to the lower abdominal area close to the urinary bladder. Median 50% threshold (T50) was determined by the up-and-down method. The values are expressed as a mean ±S.E.M. and compared by a 1-way ANOVA (Treatment) followed by Tukey *post hoc* test for multiple comparisons. *N* = 8/group. ^∗^*p* < 0.05, ^∗∗^*p* < 0.01, ^∗∗∗^*p* < 0.001 vs. control group.

Before CYP injection, the von Frey test showed no difference between the different groups (saline: 0.37 ± 0.14 g, PGB: 0.34 ± 0.08 g, CYP: 0.48 ± 0.16 g, CYP/PGB: 0.38 ± 0.10 g, data not shown). In the group receiving adjuvant and saline injection (control group), T50 scores (0.43 ± 0.16 g) were comparable to those obtained before animals were injected. Cyclophosphamide treatment induced a significant decrease in the von Frey scores (0.006 ± 0.001 g, *p* < 0.05 vs. control group). Subcutaneous acute PGB administration led to a marked significantly increase in the T50 score in healthy mice (1.29 ± 0.11 g, *p* < 0.001 vs. control group) and in CYP-treated animals (1.14 ± 0.12 g, *p* < 0.001 vs. control group) (Figure 1).

### Effect of Pregabalin on Inflammatory Parameters in Acute Cyclophosphamide-Induced Cystitis

**FIGURE 2:**
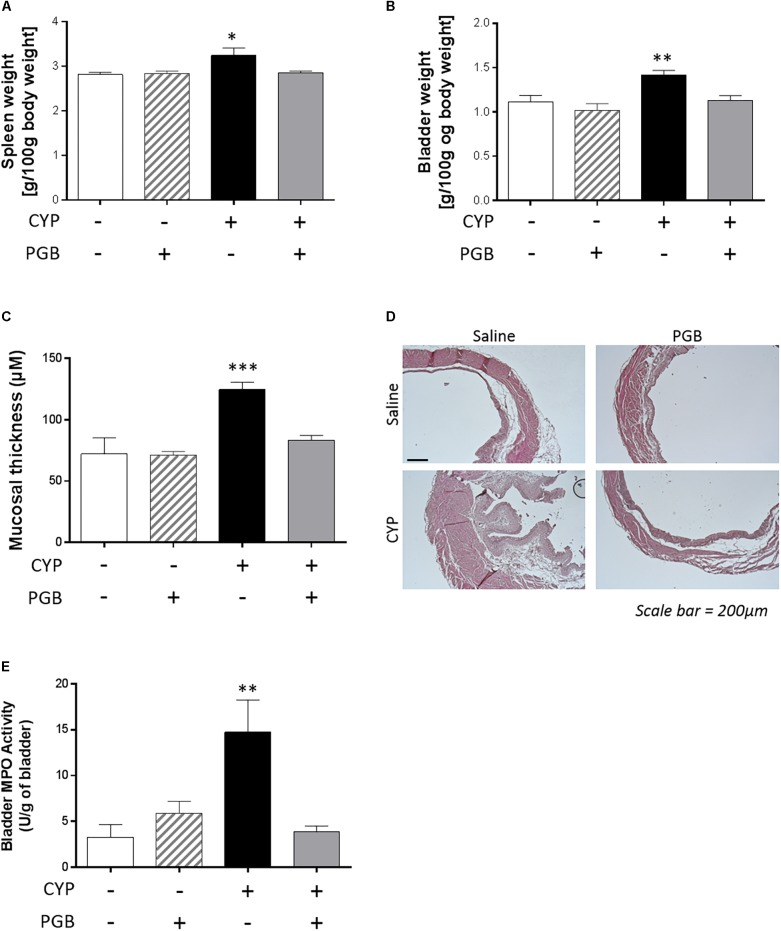
Effect of PGB (30 mg kg^-1^, s.c.) on spleen **(A)** and bladder **(B)** weight, mucosal thickness **(C,D)** and bladder MPO activity **(E)** in cyclophosphamide (CYP)-induced cystitis in mice. The values are expressed as a mean ±S.E.M. and compared by a 1-way ANOVA (Treatment) followed by Tukey *post hoc* test for multiple comparisons. For panels **A**, **B**, **E**, the result represented *n* = 8 animals / group and for panel **C**, 4 measured per section and 3 sections per animals were analyzed. ^∗^*p* < 0.05, ^∗∗^*p* < 0.01, ^∗∗∗^*p* < 0.001 vs. control group.

Cyclophosphamide induced a significant increase in the weight of the spleen (3.24 ± 0.17 g/100 g body weight, *p* < 0.05 vs. control group) and bladder (1.42 ± 0.05 g/100 g body weight, *p* < 0.01 vs. control group). This increase was not reproduced when animals were treated with PGB (spleen weight: 2.85 ± 0.04 g/100 g body weight; bladder weight: 1.13 ± 0.06 g/100 g body weight, ns, vs. control group) (Figure 2A,B).

The cyclophosphamide treatment induced a significant increase in mucosal thickness of the bladder (124.50 ± 6.21 μM, *p* < 0.001, vs. control group). In contrast, PGB acute treatment prevented bladder thickening (83.55 ± 3.60 μM, ns, vs. control group) (Figure 2C). The deleterious impact of CYP on bladder structure and the beneficial effect of PGB treatment were confirmed in a morpho-anatomicalobservation (Figure 2D).

Cyclophosphamide treatment induced an increase in bladder MPO activity (14.76 ± 3.49 U/g of bladder, *p* < 0.01, vs. control group). In contrast, PGB-treated animals had normal levels of MPO activity (3.85 ± 0.64 U/g of bladder, ns, vs. control group) (Figure 2E).

Local (bladder) and systemic (plasma) markers of inflammation (IL-6, KC, TNFα cytokines) presented the same profile with an increased level in animals receiving CYP and treated with saline, and normal levels in animals receiving CYP and treated with PGB (Table 1).

**Table 1:** Effect of pregabalin (30 mg kg^-1^, s.c) on IL-6, KC and TNFα concentration in bladder and plasmatic level in cyclophosphamide (CYP)-induced cystitis mice model.

	Cytokines	Saline/Saline (*n* = 8)	Saline/PGB (*n* = 8)	Cyp/Saline (*n* = 8)	CPY/PGB (*n* = 8)
**Bladder** pg/ml/mg of bladder	IL-6	181,3 ± 11,5	190,4 ± 23,1	300,5 ± 40,2**	166,2 ± 181,1
	KC	772,6 ± 98,1	743,9 ± 91,8	1454,1 ± 215,2**	210,6 ± 37,4*
	TNFα	135,6 ± 7,5	146,4 ± 16,5	244,9 ± 30,3***	117,5 ± 13,6
**Plasmatic** pg/ml	IL-6	1473,8 ± 10,7	1627,6 ± 67,4	2942,9 ± 78,4***	1461,5 ± 58,9
	KC	453,1 ± 65,7	289,1 ± 31,6	838,9 ± 105,4**	491,7 ± 76,9
	TNFα	999,5 ± 334,8	728,7 ± 57,6	2098,9 ± 331,6***	927,6 ± 101,8


*The values are expressed as a mean I’S.E.M. and compared by a 1-way ANOVA (Treatment) followed by Tukey *post hoc* test for multiple comparisons. *N* = 8/group. ^∗^*p* < 0.05, ^∗∗^*p* < 0.01, ^∗∗∗^*p* < 0.001 vs. control group.*

### Effect of Pregabalin on Membrane Addressing α_2_δ-1 Subunit

**FIGURE 3:**
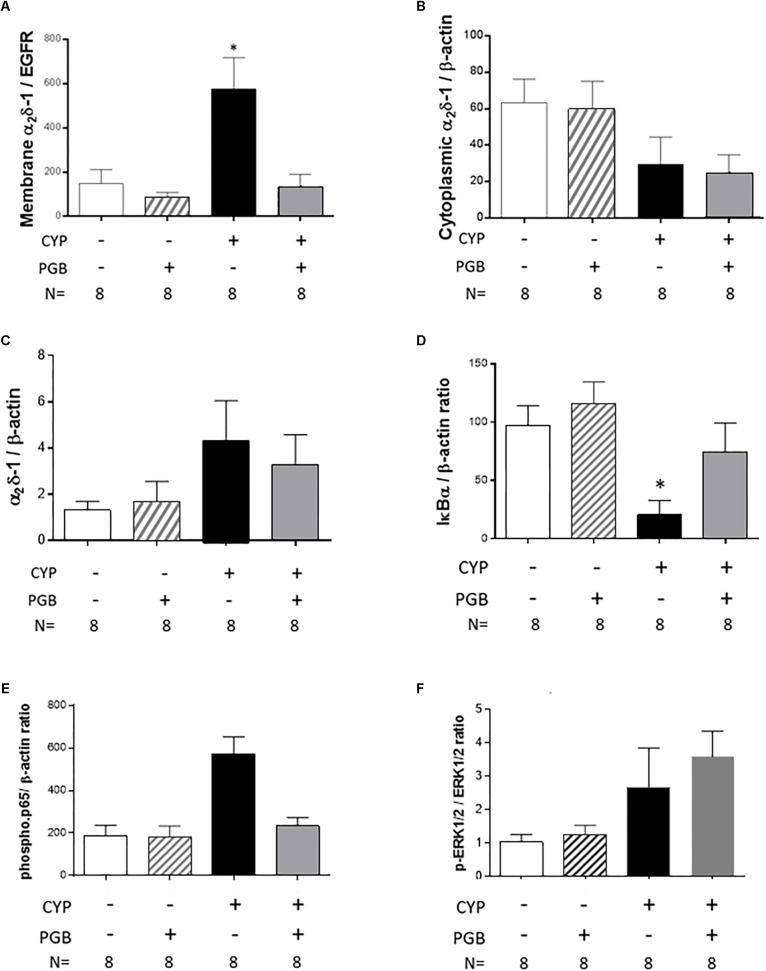
Effect of PGB (30 mg kg^-1^, s.c.) on membrane addressing α_2_δ-1 subunit and on NF-κB pathway activation in cyclophosphamide (CYP)-induced cystitis in mice. The expression of α_2_δ-1 subunit was evaluated by western blot on cytoplasmic membrane **(A)**, cytoplasm **(B),** and total cell **(C)** level. The NF-κB pathway activation was evaluated by the semi quantification of lkBα **(D)** and phospho-p65 **(E)**. The phospho-ERK1/2 pathway activation was also evaluated by the semi quantification ERK1/2 and phosphor-ERK1/2 and the ratio between these two forms was calculated **(F)**. The values are expressed as a mean ±S.E.M. and compared by a 1-way ANOVA (Treatment) followed by Tukey *post hoc* test for multiple comparisons. *N* = 8/group. ^∗^*p* < 0.05, ^∗∗^*p* < 0.01, ^∗∗∗^*p* < 0.001 vs. control group.

Cyclophosphamide induced a significant increase in membrane expression of α_2_δ-1 subunit in the bladder of the CYP-treated animals (575.50 ± 141.80, *p* < 0.05, vs. control group) (Figure 3A). The membrane expression of α_2_δ-1 subunit in animals receiving CYP and treated with PGB was similar to that in control animals (131.80 ± 58.44, ns, vs. control group) (Figure 3A). No differences were observed between the groups in α_2_δ-1 subunit cytoplasmic expression (Figure 3B) and in total α_2_δ-1 subunitexpression (Figure 3C).

### Effect of Pregabalin on NF-kB Pathway Activation in Bladder

Cyclophosphamide induced a strong and significant decrease in IkBα expression in the bladder of saline-treated animals (20.63 ± 12.45, *p* < 0.05, vs. control group) but not in that of PGB-treated mice (74.29 ± 25.05, ns, vs. control group) (Figure 3D). Concomitantly, PGB treatment blocked the significant increase in phospho-p65 expression in the bladder of mice receiving cyclophosphamide (234.20 ± 39.07, ns, vs. control group) (Figure 3E). Since a lot of other signaling pathways are involved in inflammation, we have checked the phospho-ERK1/2 pathway. Similarly, as for the phospho-p65, cyclophosphamide induced an increase in phospho-ERK1/2 expression in the bladder of saline-treated animals, and a PGB treatment did not change the level of phospho-ERK1/2 expression in cyclophosphamide-treatedmice (Figure 3F).

### Effect of Pregabalin on LPS-Induced α_2_δ-1 Expression, NF-kB Pathway Activation, Cytokine Production and Intracellular Calcium Increase on Peritoneal Exudate Cell Culture

**FIGURE 4:**
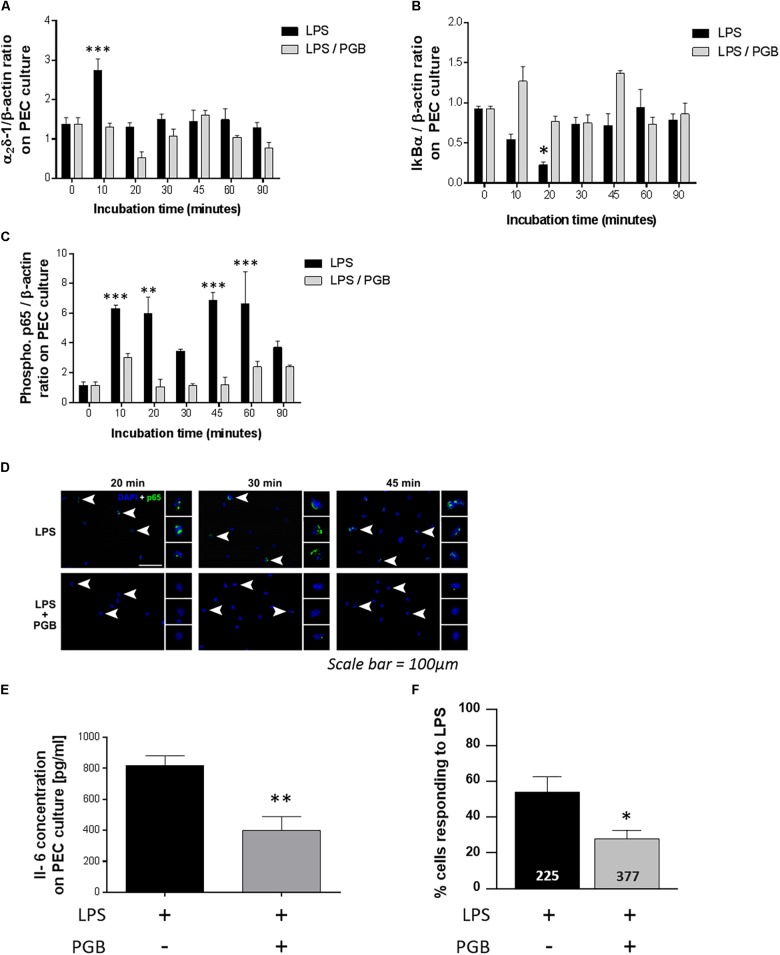
Effect of PGB (11.3 μM) on α_2_δ-1 subunit **(A)** IκBα **(B),** and phospho-p65 **(C)** expression in resident peritoneal exudate cells (PECs) stimulated with LPS (100 ng ml^-1^). The expression was measured 10, 20, 30, 45, 60, and 90 min after LPS with or without PGB treatment by western blot analyses (*n* = 4/condition). Intracellular localization of p65 in LPS-stimulated (100 ng ml^-1^) PEC treated or not with PGB (11.3 μM) was visualized by immunohistochemistry. Arrowheads indicate cells which are magnified in side panels (scale bar: 100 μm). **(D)** Effect of PGB on IL-6 level in resident PECs stimulated with LPS 24 h after these treatments (*n* = 4/group). **(E)** Percentage of LPS-stimulated PECs having [Ca^2+^]_i_ increase when incubated with saline solution or PGB (11.3 μM). **(F)** A single PEC was considered as a responder if the F340/F380 ratio for a single PEC increased by 0.05. The number of analyzed cells is shown at the bottom of the histograms (225 cells for the LPS condition and 377 cells for the LPS + PGB condition). The values are expressed as a mean ±S.E.M. and compared by a 2-way ANOVA (Model, Treatment) followed by Bonferroni *post hoc* test for multiple comparisons for α_2_δ-1, IκBα and phospho-p65 ^∗^*p* < 0.05, ^∗∗^*p* < 0.01, ^∗∗∗^*p* < 0.001 vs. t0 and by a 1-way ANOVA (Treatment) followed by Tukey *post hoc* test for multiple comparisons for IL-6 and intracellular calcium concentration analyses ^∗^*p* < 0.05, ^∗∗^*p* < 0.01, ^∗∗∗^*p* < 0.001 vs. LPS treated group.

In PEC culture, LPS treatment induced a significant increase in α_2_δ-1 expression blocked by PGB treatment (Figure 4A).

LPS induced a decrease in IkBα expression (Figure 4B) and an increase in phospho-p65 expression (Figure 4C) in LPS-stimulated PECs blocked by PGB treatment.

The increase in p65 subunit was located in the nucleus of the cells, as shown by detection of the p65 subunit by nucleus marker (DAPI). This localization was not observed following PGB treatment (Figure 4D).

The increased production of IL-6 cytokine by PECs stimulated with LPS was significantly reduced after PGB treatment (Figure 4E) as was KC cytokine production(data not shown).

As α_2_δ-1 ligands inhibit activation of VGCCs, we decided to further investigate the cellular mechanisms of PGB by measuring intracellular [Ca^2+^] in cultivated PECs with a Fura-2 probe. In three independent experiments, stimulation with LPS induced an increase in intracellular Ca^2+^ concentrations in a total of 126 out of 225 PECs (53.3 ± 9.3%). Pre-incubation with PGB significantly reduced the number of PECs having an intracellular [Ca^2+^] rise in response to LPS to 105 out of 377 (21.2 ± 3.6%, *p* < 0.05 vs. LPS group) (Figure 4F).

## Discussion

Alpha 2 delta ligands, developed as anticonvulsants and used to treat neuropathic pain, exerted a potent anti-hypersensitive and anti-inflammatory effect in a murine cystitis model. We clearly show that CYP treatment induced an increase in bladder sensitivity and inflammation that is blocked by PGB treatment. The marked decrease in cytokine overexpression induced by PGB could be due to reduced activation of theNF-kB pathway.

Cyclophosphamide-induced cystitis is a widely used model to assess bladder inflammation and related pain (Lantéri-Minet et al., 1995). Using this model, Boudes et al. (2011) performed von Frey stimulation and, as in our study, found increased sensitivity in the lower abdominal area. We first showed that PGB treatment greatly increases von Frey scores in CYP-treated animal, evidence that this anticonvulsant drug has an antinociceptive effect. To our knowledge, this is the first time that preclinical data show a potential benefit of using α_2_δ ligands in an experimental IC model. One previous preclinical study failed to find any effect of GBP in rat or mice cystitis models (Rudick et al., 2009). However, these preclinical results were surprising according the reports of its efficacy in treatment of IC (Hansen, 2000; Sasaki et al., 2001). Another clinical study reported the beneficial effects of a mixed treatment using GBP, amitryptiline and non-steroidal anti-inflammatory agents on bladder pain syndrome (Kwon et al., 2013). The antinociceptive effect of α_2_δ ligands is not surprising given that these drugs are frequently used in the treatment of chronic pain, mainly neuropathic (Verma et al., 2014) but also in fibromyalgia (Traynor et al., 2011). There is some evidence of their efficacy in the treatment of visceral pain, notably in IBS patients (Gale and Houghton, 2011). Their effect does not seem to involve a GABAergic mechanism but could result from their blockage of VGCC activation. In fact, α_2_δ ligands bind to the α_2_δ subunit exclusively expressed by high-voltage gated channels (Cheng and Chiou, 2006). Of the four isoforms, PGB seems to exhibit greater affinity for α_2_δ-1 subunits expressed in the peripheral and central nervous systems (Cheng and Chiou, 2006). PGB has a better affinity for these units than GBP, another α_2_δ ligand (Taylor, 2009) several findings suggest an involvement of these subunits in the context of visceral pain at a peripheral level in intestinal primary afferent fibers (Needham et al., 2010), or at a central level (Liao et al., 2010). Blockage of α_2_δ subunits at both peripheral and central levels could explain their antinociceptive effect in our cystitis model. However, another peripheral mechanism could be involved. Our study clearly showed that PGB is able to decrease several signs of bladder inflammation, a property that, to our knowledge, has never been mentioned in any of the preclinical models of inflammatory pain in which α_2_δ ligands were tested(Hurley et al., 2002).

The key mediator of pro-inflammatory mediator production and inflammation triggering is NF-kB (p65) (Hayden et al., 2006). Given that GBP or PGB binding on α_2_δ subunits is able to inhibit this factor in neuroblastoma and glioma cells (Park et al., 2008), the anti-inflammatory effect of PGB could be due to the blockade of NF-kB pathway activation. In the CYP model or in an *in vitro* model of PEC culture acute administration of PGB can prevent IkBα (main negative regulator of NF-kB pathway) degradation, and p65 phosphorylation (the mark of nuclear translocation of this transcription factor). A decrease in NF-kB pathway activation could result from a decrease in intracellular calcium [Ca^2+^]i induced by blockade of α_2_δ subunits. An increase in [Ca^2+^]i is important for the activation of intracellular function as transcriptional control (Mandeville and Maxfield, 1996) to promote NF-kB activation and subsequently the expression of genes involved in inflammatory responses (Martin et al., 2006). While the major Ca^2+^ entry pathway in non-excitable cells involves store-operated calcium channels, recent works also suggest that functional VGCCs are expressed in cells such as dendritic cells and macrophages (Gupta et al., 2009). Here, we propose that PGB induces a blockage in α_2_δ-1 subunit membrane expression thereby reducing the number of PECs that respond to LPS by increasing [Ca^2+^]i.

## Conclusion

To conclude, our study shows that α_2_δ ligands can reduce bladder hypersensitivity in a cystitis model and therefore is of potential benefit in patients with bladder pain. Our findings also suggest that α_2_δ ligands possess anti-inflammatory properties that contribute to their beneficial effect. We showed that the mechanism of this anti-inflammatory effect involves a decrease in α_2_δ-1 membrane expression, in [Ca^2+^]i and in NF-kB pathway activation. Clinical studies in patients with bladder pain syndrome are needed to confirm these preclinical data.

## Author Contributions

LB was involved in protocol and project development, collected and analyzed the data, and wrote the manuscript. SM collected and analyzed the data. MM, YA, and BS were involved in protocol and project development. AL, LU, and FR collected the data. FC and DA were involved in protocol/project development, the analysis of data, and wrote the manuscript.

## Conflict of Interest Statement

The authors declare that the research was conducted in the absence of any commercial or financial relationships that could be construed as a potential conflict of interest.
